# Modulating Prostate Cancer Therapy Through the Gut Microbiome: A Comprehensive Review

**DOI:** 10.3390/cancers17233842

**Published:** 2025-11-29

**Authors:** Mohammed A. Magashi Ali, Sarki A. Abdulkadir

**Affiliations:** 1 Institute of Internal Medicine, Faculty of Medicine, University of Debrecen, 4032 Debrecen, Hungary; 2 Department of Urology, Northwestern University Feinberg School of Medicine, Chicago, IL 60611, USA; 3 Robert H. Lurie Comprehensive Cancer Center, Northwestern University Feinberg School of Medicine, Chicago, IL 60611, USA

**Keywords:** prostate cancer, gut microbiome, dysbiosis

## Abstract

**Background/Objectives**: There is growing interest in the gut microbiome’s role in cancer, particularly its influence on prostate cancer therapy. This review explores how the gut microbiota modulates treatment outcomes and how prostate cancer therapies affect microbial composition. **Methods**: A semi-systematic PubMed search was performed for English-language articles published between 2010 and 2025 using relevant keywords related to prostate cancer therapy and the gut microbiome. Both original research and reviews were included, with additional studies identified through citation tracking. **Results**: The literature reveals a dynamic, bidirectional relationship between the gut microbiome and prostate cancer therapies. Gut microbes can modulate treatment efficacy and toxicity through immune regulation, metabolic activity, and the production of bioactive compounds such as short-chain fatty acids and tryptophan derivatives. These interactions influence responses to androgen deprivation therapy, chemotherapy, radiotherapy, and immunotherapy. In parallel, prostate cancer treatments induce notable shifts in gut microbial composition, reducing diversity, increasing intestinal permeability, and promoting dysbiosis. These changes may impair therapeutic outcomes. Specific microbial taxa, including *Akkermansia muciniphila*, *Faecalibacterium*, and *Bacteroides*, have been linked to both therapeutic response and microbiome alterations. **Conclusions**: The reciprocal influence between gut microbes and prostate cancer therapies presents a compelling avenue for therapeutic innovation. However, current knowledge is largely derived from preclinical or cross-cancer studies, highlighting a major evidence gap in prostate-specific research. Bridging this gap through well-designed translational studies could inform clinical strategies that harness microbiome modulation to enhance treatment efficacy, reduce toxicity, and personalize prostate cancer therapy.

## Simple Summary

The gut microbiome has become a major focus in medical research due to its wide-reaching effects on human health. This review explores its emerging connection with prostate cancer treatment, how gut bacteria may influence therapy effectiveness, and how treatment may disrupt microbial balance. By examining this two-way relationship, this review highlights new possibilities for improving cancer treatment through microbiome-based strategies. Researchers and clinicians alike may benefit from a better understanding of this evolving frontier.

## 1. Introduction

Prostate cancer is the most common cancer in men, accounting for approximately 30% of new male cancer cases in the United States in 2025 [1]. Despite its high incidence, it remains the second leading cause of cancer-related death among men [1]. Globally, prostate cancer ranked fourth in incidence and eighth in mortality in 2022, with over 1.4 million new cases and nearly 400,000 deaths reported worldwide [2]. Its incidence rose markedly in the early 1990s due to improved detection with prostate-specific antigen (PSA) [1]. Notably, incidence rates declined over the past decade. This was largely due to reduced screening following recommendations by the US Preventive Services Task Force against routine PSA testing in men aged 75 years and older in 2008 and in all men in 2012 [3].

Prostate cancer treatment spans a spectrum of radicality. Options range from invasive approaches such as prostatectomy or radiotherapy to less invasive pharmacological treatments like androgen deprivation therapy (ADT), or chemotherapy, and even conservative strategies such as active surveillance [4,5]. Despite this wide range of options, prostate cancer continues to pose significant challenges. Although mortality rates are relatively low, the 5-year survival rate for metastatic prostate cancer remains only 30%, even with newer therapeutic methods [6]. Additionally, treatment is associated with numerous side effects, including sexual dysfunction, changes in body composition, cognitive problems, and decreased quality of life [7].

Moreover, the transition from hormone-sensitive prostate cancer (HSPC) to castration resistant prostate cancer (CRPC) may partly be explained by reduced ADT-induced immunosurveillance, which depends on the presence of immunostimulatory gut bacteria [5]. Despite significant innovations in treatment, advanced cases remain largely untreatable. While seeking better treatments, perhaps we should also focus on prevention and strategies to modulate treatment responses.

The idea of manipulating the gut microbiome (GM) for health benefits dates back to the early 1900s, when Russian scientist Eli Metchnikoff hypothesized that ingesting fermented milk products containing *Lactobacillus bulgaricus* could displace harmful gut bacteria and promote longevity [8]. More recently, the Human Microbiome Project, which mapped over 1300 gut microbial strains, has deepened our understanding of the microbiome’s vital role in health and disease [9].

Several studies suggest that the GM may influence prostate cancer drug concentrations and treatment responses. Additionally, established prostate cancer risk factors such as obesity may exert their effects, in part, through gut dysbiosis [10,11]. Given these findings, it seems necessary to further explore and discuss the association between prostate cancer and the GM.

## 2. Materials and Methods

### 2.1. Literature Search

This literature review followed a semi-systematic approach designed to ensure transparency in literature selection while allowing the integration of mechanistic, preclinical, and clinical evidence. The objective was to examine original research and review articles on the role of the gut microbiome in modulating prostate-cancer therapy.

A structured search of PubMed was conducted for English-language articles published between January 2010 and January 2025. The initial search string included the following keywords: (“prostate cancer therapy” OR “prostate cancer chemotherapy” OR “prostate cancer radiotherapy” OR “prostate cancer immunotherapy”) AND (“gut microbiome” OR “gut microbiota”). Additional relevant terms were added iteratively as the review progressed to capture emerging concepts such as probiotics, prebiotics, focal microbiota transplantation, diet, and exercise.

Titles and abstracts of retrieved studies were screened for relevance, and the full text of eligible articles was assessed according to predefined selection criteria. When relevant discussions or findings were identified within selected papers, their cited references were retrieved and screened. Abstracts were reviewed, and full texts were assessed when necessary to extract additional insights. Both review articles and original studies were included if they contributed meaningful mechanistic, preclinical, or clinical perspectives to the topic. Reference lists of selected papers and recent reviews were also manually screened to identify additional relevant studies.

### 2.2. Study Selection Criteria

Due to the relatively limited number of studies directly addressing the gut microbiome in the context of prostate cancer therapy, a broad inclusion approach was adopted. Eligible studies included peer-reviewed, English-language articles that examined any aspect of the gut microbiome’s relationship to prostate cancer treatment, encompassing both original research (clinical, preclinical, or mechanistic) and relevant review articles.

Screening was performed in two stages: titles and abstracts were reviewed for relevance, followed by full-text evaluation. Non-peer reviewed and non-English language studies were excluded.

This semi-systematic method provided a structured yet flexible framework suitable for synthesizing diverse mechanistic and translational evidence across preclinical and clinical domains.

## 3. Overview of the Gut Microbiome

The typical healthy individual’s GM is made up of trillions of microbes, including bacteria, archaea, fungi, and viruses. Additionally, two healthy individuals may have completely different microbiota, as their composition is influenced by gender, community type, age, diet, geography, and even whether they were ever breastfed [8]. Research has linked the GM to various conditions, including diabetes, Alzheimer’s disease, inflammatory bowel disease, ulcerative colitis, and Crohn’s disease [12,13,14]. A bidirectional relationship between the liver and the GM also exists, mediated by the portal vein [15]. While conditions like liver disease have well-defined gut connections, the prostate is anatomically distant from the gut. As a result, the precise mechanisms linking the GM to prostate cancer remain unclear [16].

Beyond composition, the GM plays a crucial role in maintaining host health. It influences multiple aspects of host physiology, including metabolism, immunity, inflammation, and even cancer development. For one, dietary patterns and obesity, both well-established risk factors for PCa, can reshape the GM, thereby altering immune responses, metabolic processes, and inflammatory pathways [4]. Several human studies have also shown that a high-fat diet (HFD) increases anaerobic bacteria and *Bacteroides*. The higher incidence of prostate cancer in Western countries compared to Asian countries has been attributed to high-fat diets and obesity, in contrast to the different dietary patterns found in Asia [16]. The microbiome can also influence cancer development and responses to therapy through both direct and indirect mechanisms. Therapeutic modulation by the GM appears to depend on treatment modality. Commensal gastrointestinal (GI) microbes have been shown to prime tumor-associated innate immune cells to release pro-inflammatory cytokines such as tumor necrosis factor (TNF) and interleukin-12 (IL-12), thereby enhancing antitumor responses to chemotherapy and immunotherapy in preclinical models [17]. Although demonstrated in non-prostate tumor systems, these immune-mediated pathways illustrate how microbiota-driven cytokine signaling may shape therapeutic outcomes and immune tone in prostate cancer.

Reduced microbial diversity, such as that caused by antibiotic use, can promote the overgrowth of bacteria that increase intestinal permeability, inflammation, and neoplastic changes. For example, a study by Boursi et al. showed that certain antibiotics were associated with an increased risk of prostate cancer [18]. Additionally, gut bacteria have been implicated in colorectal and breast cancer through both estrogen-dependent and estrogen-independent pathways [19,20]. The concept of a functional estrobolome, a set of intestinal bacterial genes responsible for estrogen metabolism, helps explain this relationship. According to Plottel and Blaser, microbial β-glucuronidase and β-glucuronide activities regulate the conjugation and deconjugation of estrogens, thereby controlling their enterohepatic circulation [21]. When this balance is disturbed, deconjugated estrogens are reabsorbed, raising systemic concentrations. Sha et al. reported that patients with prostate cancer exhibit higher serum estrogen levels than healthy controls and that excess estrogen may promote carcinogenesis by activating polycyclic aromatic hydrocarbons (PAHs) [22]. The resulting diol-epoxide and radical-cation metabolites can form DNA adducts and cancer-promoting mutations [23]. Comparable findings have been observed in other malignancies; in advanced melanoma, antibiotic exposure was associated with reduced progression-free survival among patients treated with immune-checkpoint inhibitors, suggesting that GM disruption may compromise immunotherapy efficacy [24].

Disruptions in this balance, known as dysbiosis, have been associated with a range of diseases. Dysbiosis is any change in the GM that adversely affects the health of the host organism. It can cause endotoxinemia and increase gut permeability [25], and has been shown to activate tumor inflammation and promote tumor growth in mouse models. In prostate tissue, inflammation has been linked to the production of interleukin-6 (IL-6) and interleukin-2 (IL-2), cytokines strongly implicated in prostate-cancer pathogenesis. Persistent inflammatory signaling impairs natural-killer-cell surveillance and generates highly reactive oxygen and nitrogen species, leading to oxidative DNA damage and repeated cycles of tissue injury and regeneration. This process results in proliferative inflammatory atrophy (PIA), a lesion that can progress to high-grade prostatic intraepithelial neoplasia (PIN), a known precursor of prostate cancer. Chronic inflammation may promote morphological and molecular alterations that favor tumor initiation and progression [26]. Obesity and HFD, previously discussed as major risk factors for PCa, also drive dysbiosis. Studies have demonstrated that decreased microbial diversity is linked with systemic diseases [13]. It is therefore not surprising that the GM is considered a regulatory factor in human health, with a bidirectional relationship in which dysbiosis can promote cancer, and cancer can, in turn, alter the microbiota [22]. Recent comparative studies have revealed distinct compositional differences between the GM of healthy men and those with prostate cancer. Patients with prostate cancer generally exhibit reduced microbial diversity and a relative depletion of beneficial commensals such as *Bifidobacterium* and *Faecalibacterium*, alongside enrichment of pro-inflammatory taxa including *Bacteroides*, *Prevotella*, and *Ruminococcus* species, as summarized in Table 1. These alterations have been linked to heightened systemic inflammation and metabolic dysregulation, suggesting that dysbiosis may contribute to tumor initiation and progression rather than arise as a secondary consequence of malignancy [12,16]. Although no causal relationship has been established between PCa and GM, a study by Huang et al. showed a declining trend in GM diversity in patients with PCa [12].

## 4. Biological Mechanisms of Gut Microbiota Influence on Therapy

The gut microbiome can influence prostate-cancer progression and therapeutic response through several biological pathways, including immune modulation, androgen biosynthesis, drug metabolism, metabolic regulation, and inflammation (Table 2).

### 4.1. Immune System Modulation

One important mechanism by which the GM influences prostate cancer progression and therapy response is through modulation of the host immune system. One such pathway involves its association with HFDs, which are linked to reduced levels of short-chain fatty acids (SCFAs). SCFAs act as signaling molecules by binding to G-protein-coupled receptor 41 (GPR41, also known as free fatty acid receptor 3, FFAR3), G-protein coupled receptor 43 (GPR43 also known as free fatty-acid receptor 2, FFAR2), and hydroxycarbolic acid receptor 2 (HCAR2, also known as GPR109A), which are expressed on intestinal, immune, and endothelial cells.

Activation of these receptors triggers phosphorylation cascades involving MAPKs (ERK, JNK, and p38 MAPK), leading to suppression of NF-κB-dependent transcription and a reduction in the expression of pro-inflammatory mediators such as TNF-α, IL-1β, IL-6, and IL-8. In parallel, SCFAs exert epigenetic effects by inhibiting class I and IIa histone deacetylases, further attenuating NF-κB signaling and promoting an anti-inflammatory phenotype [35,36].

SCFA depletion due to HFDs may therefore disrupt IGF signaling, which contributes to tumor growth pathways in PCa [37]. SCFAs can stimulate systemic and local prostatic insulin-like growth factor 1 (IGF-1), thereby activating downstream signaling pathways in prostate cancer cells and promoting tumor effect [37].

Beyond IGF-1 mediated signaling, prostate cancer progression is strongly supported by activation of the phosphatidylinositol 3-kinase (PI3K), protein kinase B (AKT), and mechanistic target of rapamycin (mTOR) pathway, a major pro-survival axis frequently upregulated in advanced disease. PI3K-generated phosphatidylinositol (3,4,5)-triphosphate (PIP3) recruits and activates AKT, which then phosphorylates downstream effectors that regulate proliferation, metabolism, and resistance to apoptosis. mTOR, acting through the mTOR complex1 (mTORC 1) and mTOR complex 2 (mTORC 2), further enhances protein synthesis, lipid metabolism, and cell growth. Hyper-activation of this pathway, often driven by loss of the tumor suppressor phosphatase and tensin homolog (PTEN) or AKT and mTOR dysregulation, facilitates the transition to castration-resistant prostate cancer by compensating for suppression of androgen receptor signaling. In addition, AKT activation following chemotherapeutic stress promotes anti-apoptosis signaling and contributes to reduced sensitivity to docetaxel. Together, PI3K-AKT-mTOR represents a central adaptive mechanism that links metabolic support, androgen independence, and therapeutic resistance in prostate cancer [38].

In contrast, *Akkermansia muciniphila* has been reported to activate CD8+ T lymphocytes and enhance T-cell effector functions by upregulating IFN-yγ and granzyme B (GZMB) in both in vitro and in vivo studies, indicating a tumor-suppressive role [27]. This species also increases the M1/M2 macrophage ratio, which is significant since M2 macrophages are typically linked to tumor promotion in PCa [28]. The proposed mechanism involves extracellular vesicle-mediated delivery of pathogen-associated molecular patterns (PAMPs) and antigens, which are recognized by pattern-recognition receptors (PRRs) on immune cells [39].

Conversely, *Cutibacterium*, the most prevalent bacterium in the prostate, stimulates the infiltration of regulatory T cells (CD4+FoxP3+) and IL-17-producing Th17 cells, thereby exerting a tumor-promoting and procarcinogenic effect [31].

### 4.2. Microbial Metabolism of Therapeutics

Irinotecan, used in colorectal and pancreatic cancers, has been suggested for prostatic small cell carcinoma (SCC) [40]. However, one of its major side effects is severe diarrhea, which results from the activation of its toxic metabolite SN-38 by microbial β-glucuronidase in the gut. Although antibiotics were initially used to suppress this enzyme, their poor safety profile and disruption of beneficial gut bacteria raised concerns.

Mahdi et al. demonstrated that mice treated with irinotecan developed mucosal inflammation, submucosal edema, and focal necrosis. Co-administration of *Lactobacillus* species (including *L. acidophilus*, *L. plantarum*, and *L. rhamnosus*) restored normal colon histology, likely due to increased mucin secretion and goblet cell counts. Moreover, mice treated with both irinotecan and *Lactobacillus* spp. were reported to show reduced TNF-α and IL-6 in the colon, suggesting a potential anti-inflammatory effect. This effect was hypothesized to result from decreased expression of caspase-3, which in turn reduced pro-inflammatory cytokines such as IL-1α and IFN-γ [33].

In line with this, Se-Hoon et al. analyzed the GM in small cell lung cancer patients and found higher response rates in those harboring *Bifidobacterium bifidum*. In mouse models, this species has been reported to enhance immune modulation, possibly through increased IFN-γ production and other metabolites [30].

### 4.3. Metabolite Production

The GM plays a critical role in the production of SCFAs, including butyrate, acetate, propionate, and isopropionate, which serve as key energy sources for gut epithelial cells. These SCFAs are not only vital for gut health but are also involved in systemic processes such as lipid, glucose, and cholesterol metabolism [16].

Notably, studies on Japanese men have shown that bacterial taxa known to produce SCFAs were more abundant in individuals with high Gleason score prostate cancer (a histopathological grading system ranging from 6 to 10 that reflects tumor differentiation and aggressiveness) compared to the general population. Interestingly, the presence of metastases did not significantly alter the concentration of these bacteria, suggesting that microbial changes may contribute to cancer development rather than result from it [16].

Antibiotic-induced depletion of SCFA-producing bacteria has been shown to suppress IGF-1 and downstream MAPK and PI3K signaling, consistent with the SCFA-mediated mechanisms described in Section 4.1.

Beyond SCFAs, other microbial metabolites such as those derived from tryptophan also play important roles in shaping the tumor microenvironment.

Tryptophan metabolism helps shape a tumor-promoting microenvironment (TME), largely by fueling signaling pathways that favor tumor growth [41]. The enzyme tryptophan hydroxylase 1 (TPH1) converts tryptophan into serotonin, which in prostate cancer cells activates growth- and survival-related signaling. Recent evidence shows that serotonin stimulates the β-catenin pathway, increasing the expression of genes that drive cell proliferation and migration. β-catenin, together with the transcription factor ZBP-89, further enhances TPH1 expression, forming a feedback loop that maintains high serotonin levels and supports tumor progression. Blocking TPH1 activity with 4-chloro-DL-phenylalanine interrupts this cycle, reducing tumor growth and strengthening the effect of paclitaxel in prostate-cancer models [42].

Additionally, the aryl hydrocarbon receptor (AHR), which can be activated by tryptophan-derived ligands, contributes to an immunosuppressive and tumor-permissive environment. Certain gut microbes, including *Clostridium sporogenes* and *Ruminococcus gnavus*, have been identified as sources of these AHR ligands [29], highlighting a potential link between GM and tryptophan-mediated tumor modulation.

### 4.4. Intestinal Permeability and Inflammation

As previously discussed, HFDs have been linked to an increased incidence of prostate cancer. Less commonly addressed is their role in reducing the alpha-diversity of the GM, a measure of microbial richness and evenness associated with a healthy and resilient gut environment. HFDs have been reported to increase the abundance of anaerobic bacteria and *Bacteroides* in the gut. These changes in GM can increase the translocation of Gram-negative bacteria into the bloodstream and mesenteric fat tissue via the intestinal mucosa, ultimately leading to chronic inflammation [12].

Chronic inflammation is a well-established driver of dysbiosis and cancer risk. Several studies have reported an increased incidence of prostate cancer among men with a history of prostatitis [22]. Notably, a study by Poutahidis et al. demonstrated that gastrointestinal bacterial infection alone was sufficient to induce PIN and microinvasive carcinoma in mice [43]. Supporting this, Liss et al. collected rectal swabs from patients undergoing transrectal prostate biopsy and found that those with prostate cancer exhibited increased levels of pro-inflammatory bacteria such as *Bacteroides* and *Streptococcus* [44].

The mechanistic link between inflammation and carcinogenesis involves oxidative stress and tissue remodeling. During inflammation, immune cells release reactive oxygen and nitrogen species, which can directly damage DNA and surrounding cellular structures. This oxidative damage may trigger a cascade of repair processes, angiogenesis, and uncontrolled cellular proliferation. The result is proliferative inflammatory atrophy (PIA), which is considered a precursor to PIN and potentially adenocarcinoma [22].

Parallel research has also shown that certain gut microbes may contribute to this pro-inflammatory environment. For instance, *Faecalibacterium* has been reported to activate the NF-κB pathway and upregulate pro-inflammatory cytokines, including IL-23 and IL-17, which drive Th-17 cell differentiation and activation. These cytokines have been associated with tumor-promoting inflammation, possibly through STAT3 signaling [32]. The enrichment of *Prevotella*, another genus commonly found in the GM of colorectal cancer patients, has also been observed in individuals with prostate cancer, suggesting a possible shared microbial signature between the two malignancies [12]. However, the underlying mechanisms remain to be elucidated.

**Table 2:** Mechanistic pathways through which the gut microbiome influences prostate cancer progression and treatment response. These include effects on immunity, metabolism, androgen regulation, and drug interaction, each impacting different aspects of therapy and efficacy. ↑ = increased; → = no significant change.

Mechanism	Description	Implications for Prostate Cancer	Therapy Context
Immune modulation	Altered Th1/Th17/Treg balance; CD8+ T cell activation [27,28,31,39]	Enhances or suppresses anti-tumor immunity	ADT, Immunotherapy
Androgen biosynthesis	Microbial conversion of precursors (e.g pregnenolone→ DHEA/testosterone) [5,45]	Promotes CRPC and therapy resistance	ADT
Metabolic regulation	SCFA production, IGF-1 stimulation, tryptophan metabolism [16,29,37,41,42]	Influences tumor growth, immune activity	ADT, Chemotherapy
Inflammation	NF-κB activation, ↑ pro-inflammatory cytokines, dysbiosis-induced permeability [12,22,32,43,44]	Promotes carcinogenesis and reduces treatment efficacy	General, Radiotherapy, Chemotherapy
Drug metabolism (activation/inactivation)	Β-glucuronidase activation of irinotecan; microbial inactivation of gemcitabine [22,30,33,40]	Increases toxicity or decreases drug efficacyIncreases toxicity or decreases drug efficacy	Chemotherapy
Barrier integrity maintenance	SCFA-mediated mucin secretion and epithelial protection [33]	Reduces GI toxicity and systemic inflammation	Radiotherapy
Epigenetic & metabolite effects	HDAC inhibition, AHR ligands, in spine signaling [29]	Affects immune cell differentiation and function	Immunotherapy

## 5. Impact on Prostate Cancer Therapy

**Figure 1:**
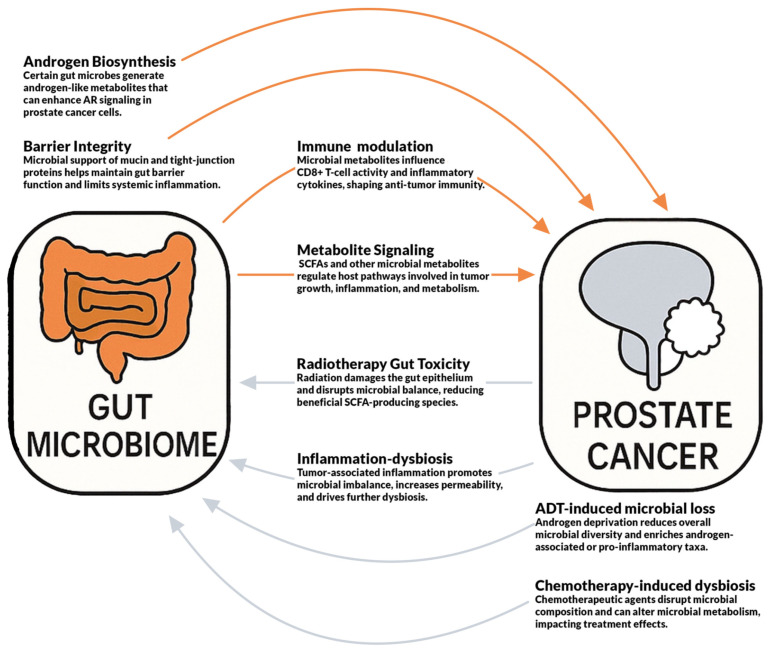
Bidirectional interactions between the gut microbiome and prostate cancer. The gut microbiota modulates prostate cancer through hormonal, immunological, metabolic, and barrier-related pathways while prostate cancer and its treatments, including radiotherapy, inflammation, androgen deprivation therapy, and chemotherapy, reshape gut microbial composition and function. The figure summarizes key feedback mechanisms influencing disease progression and therapeutic response.

The relationship between the GM and PCa is shaped by a network of bidirectional hormonal, immune, metabolic, and barrier-related interactions. Figure 1 summarizes these pathways and illustrates how microbial activity and PCa treatments influence each other. These mechanisms underpin the facets described across major therapy classes, which are outlined in Table 3 and discussed in the subsections that follow.

### 5.1. Androgen Deprivation Therapy

The gut microbiome influences treatment response across major prostate-cancer therapies through diverse mechanisms, including modulation of immune pathways, inflammation, and metabolism.

PCa is often managed through ADT, typically using androgen synthesis inhibitors such as abiraterone acetate or androgen receptor antagonists like enzalutamide. These therapies are initially effective at suppressing tumor growth in HSPC. However, many patients eventually progress to CRPC, a more aggressive and treatment-refractory form of the disease. While this transition has traditionally been attributed to cancer cell-intrinsic resistance mechanisms, emerging evidence suggests that cancer cell-extrinsic factors, particularly involving the GM, also contribute significantly. The apparent failure of ADT-induced immunosurveillance, which may depend on the presence of immunostimulatory gut bacteria, suggests that the GM could modulate therapeutic efficacy [5].

Beyond immunomodulation, the GM also plays a direct role in drug metabolism, potentially undermining the pharmacological effectiveness of ADT. Certain bacterial species possess the capacity to degrade drugs used in ADT, thereby reducing their therapeutic efficacy. Others are capable of synthesizing androgens, effectively replenishing systemic testosterone levels and undermining the goal of androgen suppression. Emerging evidence shows that men with CRPC often harbor increased levels of these androgen-producing bacteria in their GM. These findings highlight the potential influence of the GM’s composition, whether through immunostimulatory, immunosuppressive, or drug-inactivating mechanisms, on patient responses to ADT [5].

Animal studies have further illustrated the GM’s influence on ADT efficacy. In a prostate cancer mouse model, depletion of the GM using broad-spectrum antibiotics diminished the therapeutic effect of ADT. Interestingly, prostate cancer itself was shown to reduce the abundance of *Akkermansia muciniphila*, a gut bacterium known for its immunostimulatory properties, while ADT reversed this reduction. Restoring *A. muciniphila* through oral gavage or cohousing with tumor-free mice enhanced the antitumor response to ADT, suggesting a beneficial role for this species in mediating treatment efficacy [46].

However, the role of *A. muciniphila* in human prostate cancer remains controversial, as it has been shown to elicit both immune-dependent and independent anticancer effects. In abiraterone-treated patients progressing toward CRPC, *A. muciniphila* levels were found to increase alongside menaquinone (vitamin K2) biosynthesis, an immune-independent mechanism shown to inhibit prostate cancer growth in vitro. Additionally, extracellular vesicles derived from *A. muciniphila* have been reported to activate cytotoxic T lymphocytes against prostate tumors in mice. Paradoxically, one clinical study noted that patients with metastatic CRPC progressing on enzalutamide who responded to PD-1 blockade showed a decrease in *A. muciniphila*, contradicting patterns observed in other cancers such as non-small cell lung cancer, melanoma, and urothelial carcinoma, where its presence was associated with improved immunotherapy outcomes [5].

ADT itself has been implicated in altering gut microbial diversity, potentially contributing to dysbiosis and altered androgen metabolism. Specifically, patients undergoing ADT exhibit a decrease in both alpha- and beta-diversity of their gut microbial communities, metrics that reflect species richness and variation between individuals, respectively. These changes may precede or accompany the onset of dysbiosis. Notably, prior studies have shown that specific bacterial species present in human stool samples can modulate androgen metabolism, further implicating the GM in the regulation of hormone-driven prostate cancer progression [5].

Perhaps more critically, emerging research has shown that the GM from either patients with CRPC or castrated mice, can convert androgen precursors into active androgens, such as dehydroepiandrosterone (DHEA) and testosterone, which are then absorbed into the systemic circulation. Depletion of GM in mice resulted in significantly reduced circulating androgen levels, highlighting the microbiota’s role as an extragonadal source of these hormones [5].

These associations are further supported by experimental evidence from murine models, which have demonstrated a direct causal role for these microbes in modulating tumor progression and androgen levels. Microbiota ablation using broad-spectrum antibiotics reduced tumor growth and delayed the onset of CRPC. 16S rRNA gene sequencing revealed an enrichment of *Ruminococcus gnavus* and *Bacteroides acidifaciens* in the GM of castrated mice, with *R. gnavus* alone being sufficient to accelerate tumor progression [45].

Fecal microbiota transplantation (FMT) from castration-resistant mice produced a similar outcome, rapidly inducing CRPC, increasing cell proliferation, and shortening animal survival. Targeted metabolomic analyses showed elevated circulating androgen levels in both post-CR-FMT mice and those administered *R. gnavus*, confirming the microbiota’s role in extragonadal androgen biosynthesis [45].

Both *R. gnavus* and *B. acidifaciens* were found to convert steroidal precursors like pregnenolone and hydroxypregnenolone into active androgens, while also upregulating androgen receptor (AR) gene expression in CRPC cells. Microbially derived androgens such as testosterone and dihydrotestosterone (DHT) can directly activate AR signaling. Binding of these ligands to AR promotes receptor stabilization, nuclear translocation, and transcription of androgen-responsive genes that drive prostate-cancer cell proliferation and survival. Even under castrate levels of circulating androgens, sustained AR activation supports progression toward castration-resistant prostate cancer, mirroring resistance mechanisms observed with AR overexpression and intratumoral steroidogenesis [47]. Supporting the hypothesis that these intermediates may enter the gut via enterohepatic circulation, intravenous injection of deuterated pregnenolone into castrated mice led to reduced levels of deuterated androgens following microbiota ablation [45].

Conversely, certain species such as *Prevotella stercorea* appear protective, delaying CRPC onset and correlating with favorable clinical outcomes. These findings highlight the potential of microbial-based interventions to improve ADT efficacy [45].

### 5.2. Chemotherapy

An experiment by Zhong et al. demonstrated a significant interaction between the GM and the efficacy of docetaxel in prostate cancer. Treatment of mice with broad-spectrum antibiotics induced marked dysbiosis characterized by an overrepresentation of *Proteobacteria*. This alteration was accompanied by elevated lipopolysaccharide (LPS) concentrations in the serum compared to untreated controls, despite higher fecal LPS levels in non-antibiotic-treated mice. LPS, derived from Gram-negative bacteria, is known to induce inflammation through activation of the TLR4-NF-κB pathway, promoting cytokine secretion. Correspondingly, serum IL-6 levels were also increased in antibiotic-treated mice. IL-6 in turn activates STAT3 signaling in an autocrine manner, a pathway known to promote cancer progression and confer resistance to chemotherapeutic agents, including docetaxel and 5-fluorouracil [48,49]. Functional assays supported this mechanistic link: suppression of the IL-6-STAT3 pathway improved docetaxel sensitivity in prostate cancer cell lines, and in vivo experiments confirmed significantly reduced tumor volume and weight in mice treated with docetaxel, an effect that was diminished in dysbiotic mice. These findings suggest that the GM may modulate systemic inflammatory pathways that influence chemotherapy tolerance and efficacy [48].

Beyond docetaxel, other chemotherapeutics also demonstrate microbiota-dependent interactions. Cyclophosphamide, for instance, exerts part of its efficacy through increasing gut permeability, facilitating microbial translocation into secondary lymphoid organs (e.g., tonsils, lymph nodes, spleen), where these microbes enhance antitumor immune responses. In contrast, certain microbes can impair chemotherapy: *Mycoplasma hyorhinis* has been reported to metabolize gemcitabine into an inactive form, potentially reducing its anticancer activity [50].

Collectively, these findings suggest that the GM profoundly influences chemotherapy outcomes in prostate cancer, by either enhancing immune-mediated responses or diminishing drug efficacy. Targeting the microbiota through interventions such as microbiota restoration or inflammation modulation may therefore represent a strategy to optimize chemotherapy responsiveness.

### 5.3. Immunotherapy

PCa is widely considered an immunologically ‘cold’ tumor, with low tumor mutation burden, limited PD-L1 expression, minimal T-cell infiltration, and a strongly immunosuppressive TME. These features contribute to the limited efficacy of ICIs such as pembrolizumab and ipilimumab, which currently show clinical benefit only in a subset of patients with mismatch repair deficiencies, microsatellite instability-high tumors, or CDK12 mutations [50].

Preclinical and clinical studies in other cancers have suggested that the GM may enhance antitumor immunity and improve ICI responses through both innate and adaptive mechanisms. On the innate side, species like *Bifidobacterium* and *Bacteroides fragilis* promote dendritic cell maturation, IL-12-dependent Th1 responses, and macrophage polarization toward antitumor phenotypes [51,52,53]. Natural killer (NK) cell activation has also been linked to high gut microbial diversity and *Lactobacillus* supplementation in previously non-responding models [54,55]. On the adaptive side, favorable taxa such as *Clostridiales*, *Ruminococcaceae*, and *Faecalibacterium* have been associated with improved antigen presentation and enhanced effector CD4+ and CD8+ T-cell function in both the periphery and the TME [56,57].

Microbial metabolites further contribute to this effect. Inosine, produced by *Akkermansia muciniphila* and *Bifidobacterium pseudolongum*, has been reported to improve ICI efficacy by enhancing tumor antigen presentation via IFNγ and TNFα pathways, activating T cells through adenosine A2A receptor signaling, and serving as an alternative carbon source for CD8+ T cells under metabolic stress [58,59,60]. Similarly, SCFAs such as butyrate and propionate, produced by taxa, are thought to synergize with ICIs by modulating epigenetic regulation (via HDAC inhibition), upregulating cell-cycle inhibitors, and providing metabolic support to immune cells [56,61,62].

Collectively, these findings highlight the potential for microbiota-targeted interventions, such as FMT, probiotics, or engineered consortia to reprogram the TME, enhance tumor immunogenicity, and improve ICI responsiveness in PCa [63,64]. However, direct prostate-specific evidence remains limited, underscoring the need for translational and clinical studies to adapt these strategies to this context.

### 5.4. Radiotherapy

Radiotherapy (RT) remains one of the cornerstone treatments for PCa, applied either as a definitive curative approach or as an adjuvant/salvage therapy following prostatectomy, with excellent efficacy outcomes [64]. Advances in external beam RT have enabled more precise tumor targeting, improving tumor control while reducing collateral tissue damage [65].

Despite these improvements, treatment-related toxicities remain a significant concern. Between 10 and 50% of patients experience moderate to severe acute GI side effects, including proctitis, diarrhea, rectal bleeding, and abdominal pain within 90 days after RT [66]. These toxicities can compromise quality of life and, in some cases, necessitate treatment interruption [65].

Emerging evidence suggests that the GM may modulate radiation response and toxicity. Alterations in the intestinal microbiome, particularly reduced bacterial diversity, have been strongly associated with both acute and late radiation-induced GI complications [64,65]. The MARS study reported evidence supporting this association: patients who developed early GI toxicity exhibited a sustained reduction in bacterial diversity compared to those without toxicity, a pattern mirrored in larger cohorts assessed for late toxicity. Notably, an increased relative abundance of SCFA-producing bacteria was also linked to both acute and late radiation enteropathy [64,65].

SCFAs such as butyrate play an essential role in maintaining intestinal homeostasis and may protect against RT-induced injury by strengthening the mucosal barrier, enhancing mucus production, and promoting mucosal Treg cell recruitment [64]. Thus, interventions aimed at restoring microbiome diversity or augmenting SCFA production could mitigate RT-related GI injury.

Beyond overall diversity loss, radiotherapy induces specific compositional and functional shifts in the GM. Patients with toxicity demonstrated higher abundances of *Fusobacteriaceae* and *Fusobacterium*, whereas *Christensenellaceae*, *Sporobacter*, *Eubacterium eligens*, and *Bacteroides fragilis* showed differential associations with toxicity status [65]. Additionally, RT was associated with an increased *Firmicutes*-to-*Bacteroidetes* ratio, reflecting a substantial community-wide shift [34].

Functional analyses revealed that patients experiencing toxicity displayed decreasing trends in carbon fixation pathways and bacterial secretion systems, along with fluctuations in pathways related to pertussis, D-glutamine and D-glutamate metabolism, and steroid hormone biosynthesis. In contrast, patients without toxicity exhibited a sustained increase in PI3K-Akt signaling, associated with cellular survival and mucosal repair, which stabilized after treatment [65]. Importantly, several of these disrupted pathways partially recovered to pre-treatment levels within one month post-RT [65].

Microbiota-targeted interventions have shown potential for reducing radiation-induced GI injury. Administration of *Alistipes onderdonkii* in murine models has been reported to improve survival and reduce intestinal damage following irradiation, likely by alleviating oxidative stress, maintaining mucosal integrity, and enhancing resistance to injury [34]. Similarly, *Lactobacillus* species, particularly *L. reuteri* and *L. brevis*, demonstrated radioprotective effects by preventing villus shortening, reducing epithelial apoptosis, and preserving mucosal thickness [34]. Beyond specific taxa, metabolites such as butyrate, produced by commensal bacteria, reinforce the mucus layer and promote mucosal immune regulation, providing further protection against RT-induced GI toxicity [67,68].

**Table 3:** Summary of gut microbiome influence on prostate cancer therapies. The table outlines key mechanisms through which the microbiome modulates treatment response and highlights representative microbial species or metabolites associated with each therapy.

Therapy	Microbiome Influence	Key Mechanisms	Supporting Species/Metabolites
Androgen Deprivation Therapy (ADT)	Enhances or undermines therapy	Androgen biosynthesis, immune modulation	*Akkermansia* [27], *Ruminococcus gnavus* [45]
Chemotherapy	Modifies efficacy and toxicity	IL-6/STAT3 axis, drug metabolism	*Lactobacillus* spp. [33], *Mycoplasma hyorhinis* [22]
Immunotherapy	Affects ICI response	Inosine, SCFA production, antigen presentation	*Bifidobacterium* [30], *Faecalibacterium* [57], inosine [51], SCFAs [57]
Radiotherapy	Modulates GI toxicity	SCFA production, inflammation	*Alistipes onderdonkii* [34], *lactobacillus* spp. [34], butyrate [64]

## 6. Microbiome Based Therapeutic Strategies

While current treatments for PCa, including active surveillance, surgery, radiotherapy, and androgen deprivation therapy, remain the standard of care, they are not without limitations. These approaches can be hindered by patient non-compliance due to adverse side effects, the eventual development of therapeutic resistance, and the biological constraints of hormonal manipulation. These challenges highlight the urgent need for alternative or adjunctive strategies that can improve treatment efficacy, reduce toxicity, and enhance patient quality of life. Microbiome-based therapeutic interventions are being explored as a promising avenue to address these gaps (Table 4).

### 6.1. Probiotics and Prebiotics

Probiotics and prebiotics are increasingly recognized as potential adjuvants in cancer therapy. In colorectal cancer, for example, *Lactobacillus rhamnosus* GG has been administered alongside conventional treatments to support the re-establishment of commensal microbiota and alleviate GI stress. These findings may be extrapolated to prostate cancer, although direct evidence remains limited. Clinical studies assessing prebiotics across different diseases have been heterogeneous in design and have produced conflicting results, largely owing to variability in intervention type, duration, and outcome measure [69]. Moreover, most available data evaluate gastrointestinal rather than cancer-specific outcomes, underscoring the limited mechanistic and clinical understanding of prebiotics in PCa.

Importantly, probiotics have also been associated with rare but serious adverse events, such as sepsis in immunocompromised individuals, underscoring the need for further investigation into their safety and efficacy in this context [70].

In a prospective study, Akmansu et al. reported that prebiotic supplementation stabilized TNF-α levels during radiotherapy for pelvic malignancies, whereas levels increased in controls. This stabilizing effect may relate to changes in microbial composition induced by prebiotics, although mechanistic pathways remain incompletely defined. When prebiotics and probiotics were administered in combination, there was no significant increase in either IL-6 or TNF-α, suggesting a synergistic effect [71]. Similar small-scale trials in patients with pelvic malignancies, including prostate cancer, reported modest reductions in diarrhea frequency and increases in *Bifidobacterium* counts following prebiotic supplementation, but quality-of-life benefits and oncologic outcomes remain unproven [72].

Probiotics have been reported to show potential in mitigating radiation-induced gastrointestinal injury and reducing inflammatory cytokines. However, an increased incidence of urological symptoms was noted when prebiotics and probiotics were administered together, possibly related to microbiota-dependent metabolic effects rather than systemic cytokines changes [71]. Recent analyses also highlight strain-dependent variability and inconsistent durability of microbiota changes after supplementation [69]. These limitations emphasize the need for prostate-specific, mechanistically informed trials that assess not only gastrointestinal but also oncologic outcomes.

### 6.2. Fecal Microbiota Transplantation

FMT involves transferring GM from healthy donors to patients via the gastrointestinal tract to restore microbial balance. It is a well-established treatment for recurrent *Clostridioides difficile* infection and has been explored in autoimmune conditions, metabolic syndromes, multiple sclerosis, and various cancers, particularly those of the digestive system [73]. In PCa, FMT has been proposed as a therapeutic approach aimed at enhancing beneficial bacteria, reducing pathogenic species, and introducing advantageous metabolites such as SCFAs, IGF-1, and folic acid [74].

FMT can be administered through several routes: oral capsules and nasoduodenal tubes for small intestine delivery, and colonoscopy, rectosigmoidoscopy, or enemas for large intestine delivery [73]. Each method carries distinct advantages and limitations. For instance, fecal infusion via colonoscopy allows direct access but is more invasive and costly, whereas enemas are well-tolerated but may have limited efficacy. The nasoduodenal tube is often the preferred method for its balance of safety and effectiveness. However, both upper and lower GI routes carry risks, including aspiration pneumonia, vomiting, procedural discomfort, and, rarely, severe complications such as perforation, bacteremia, sepsis, multi-organ failure, or death [73].

Although FMT is generally regarded as safe, its application in prostate cancer remains investigational, as no clinical trials or systematic reviews have yet evaluated its efficacy or safety in this context. Much of the current understanding derives from gastrointestinal and metabolic disorders, which limits direct translation of PCa. General clinical data indicate that adverse events are predominantly mild and gastrointestinal, including transient diarrhea, bloating, and abdominal discomfort, while serious complications such as perforation, bacteremia, sepsis, multi-organ failure, and death have been documented [75]. Systematic reviews and meta-analyses estimate that serious adverse events occur in fewer than 1% of cases and may include colectomy, infection-related hospitalization, and other life-threatening complications, whereas the overall incidence of adverse events is approximately 28%, with abdominal discomfort being the most frequent [75]. Rare transmission events such as Shiga toxin-producing *Escherichia coli*, have also been reported, underscoring the importance of stringent donor screening and post-treatment monitoring [75]. These findings highlight the need for prostate-specific studies with standardized protocols, validated safety endpoints, and longer follow-up to establish whether FMT can be safely integrated into prostate-cancer management.

Beyond procedural safety, several factors influence FMT outcomes, including donor and recipient characteristics, dosing frequency, delivery route, and confounding variables such as diet, medication, and co-morbidities [73]. The regulatory landscape for FMT remains inconsistent across jurisdictions, with varying classifications ranging from biological products to medicinal or tissue-based therapy. Several countries still lack clear regulatory guidance, creating uncertainty for clinical trial design and implementation [76].

Ethical considerations extend beyond patient safety to include donor rights, informed consent, and autonomy. Donor recruitment can be difficult because the definition of an ideal or healthy donor remains uncertain, and several key factors must be considered to optimize FMT in PCa. These include patient-specific variables such as age, cancer stage, co-morbidities, and treatment adherence, as well as donor characteristics such as overall health and the absence of metabolic, autoimmune, or malignant diseases [73]. The invasive nature of repeated screening procedures raises legitimate concerns regarding privacy and donor welfare. Ongoing debate surrounds whether donors should be compensated for their participation and who bears responsibility for long-term tracking should a previously healthy donor later develop conditions of concern [76]. For PCa-specific trials, these considerations require even greater scrutiny: oncology populations must be fully informed of experimental status, potential microbiome-related risks, and the need for extended monitoring.

From a mechanistic perspective, FMT also intersects with pharmacomicrobiomics, the study of how the GM influences the pharmacokinetics (PK) and pharmacodynamics (PD) of drugs. Microbial enzymes may activate, inactivate, or detoxify therapeutic compounds, thereby influencing systemic drug levels and efficacy. Although most evidence comes from gastrointestinal and metabolic diseases, similar principles may apply to prostate-cancer therapy, where microbiota-mediated metabolism could influence androgen-deprivation or chemotherapy responses. Incorporating FMT as a microbiome-modulating strategy may therefore provide a means to optimize host-drug interactions and improve therapeutic outcomes [76].

Overall, FMT represents a promising yet experimental approach in prostate-cancer management, and its clinical applications will depend on the outcomes of future well-designed trials.

### 6.3. Diet and Lifestyle

Long-term consumption of an HFD has been associated with decreased alpha-diversity of the GM in patients with PCa [12]. Shin et al. reported that Japanese men adopting a Western-style dietary pattern, characterized by high intake of red meat, potatoes, and full-fat dairy products, exhibited a higher incidence of PCa compared to those adhering to a prudent dietary pattern rich in vegetables, fruits, and fish. Obesity, often associated with Western dietary habits, has also been consistently associated with an increased risk of PCa. Animal-derived saturated fats appear to contribute to PCa progression, whereas unsaturated fatty acids derived from fish and vegetable oils have been associated with reduced risk [77,78].

Patients undergoing ADT experience unfavorable changes in body composition, including a significant loss of lean mass and an increase in fat mass, which may negatively affect metabolic health and GM composition [11]. It has been hypothesized that the hormonal consequences of ADT contribute to gut dysbiosis, while lifestyle modifications such as dietary changes and exercise may mitigate these effects. Preliminary evidence indicates that exercise independently improves gut microbial diversity and composition, potentially enhancing systemic metabolic and inflammatory profiles [79]. Additionally, exercise has been shown to attenuate or reverse fat accumulation in PCa patients on ADT and improve lean body mass and overall body composition [80,81]. These improvements may mediate beneficial changes in gut microbiota composition, reduce systemic inflammation, and favorably influence metabolic profiles in this population [11].

Collectively, these findings suggest that diet and lifestyle modification could serve as potential adjunct strategies for modulating the GM and improving outcomes in PCa. Although observational, the results from epidemiological and interventional studies consistently support a link between dietary patterns, metabolic health, and prostate-cancer progression. While the association between diet, exercise, and prostate-cancer risk is well supported, the specific microbial and metabolic mediators of these effects remain to be fully clarified.

### 6.4. Challenges in Microbiome-Based Therapeutics

Despite the promise of microbiome-based interventions in PCa, several challenges continue to limit their clinical translation. One major limitation is the absence of standardized protocols for interventions such as probiotics, prebiotics, and FMT. Variability in donor selection, dosage, frequency, and delivery methods complicates the reproducibility of results and raises questions about safety and efficacy. For example, although probiotics and prebiotics are generally well tolerated, rare but serious adverse events such as sepsis have been reported in immunocompromised patients, emphasizing the importance of careful patient selection and monitoring [70].

FMT poses its own set of challenges. Although it has demonstrated safety in other indications, such as *Clostridioides difficile* infection, its application in PCa remains investigational, with no standardized criteria for donor screening, preparation, or administration. Additionally, the long-term effects of altering the GM through FMT are poorly understood, raising concerns about unintended metabolic or immunological consequences [67].

**Table 4:** Summary of microbiome-modulating interventions relevant to prostate cancer. This table outlines evidence-based strategies aimed at restoring or modifying gut microbial composition and function to enhance therapy outcomes or reduce toxicity across various treatment contexts.

Intervention	Mechanism	Supporting Evidence	Application
Probiotics	Restore microbial balance; reduce inflammation; enhance barrier integrity	*Lactobacillus* spp. reduced irinotecan- induced toxicity [33]; shown to prevent epithelial apoptosis post-radiotherapy	Mitigating GI toxicity during Chemo/RT
Prebiotics	Promote SCFA production; support growth of beneficial microbes	Stabilised TNF-α and IL-6 levels during pelvic RT; improved mucosal regulation via SCFAs [34]	Supportive during RT
FMT	Recolonize gut with healthy microbiota; introduce beneficial metabolites	Proposed for PCa based on success in GI cancers and Melanoma [45]; donor selection and safety under study	Experimental; potential across therapies
Dietary Modification	Modulates microbial diversity; influences inflammation and metabolism	HFD reduced alpha- Diversity [16]; Japanese prudent diet linked to lower PCa incidence [68]	Preventive and adjunct across all therapies
Exercise	Improves microbial diversity and systemic inflammation	Increased gut diversity and lean mass in ADT patients; reduced inflammatory markers [70]	Adjunct to ADT and metabolic management

Ethical and regulatory considerations further complicate the integration of these strategies into clinical practice. Establishing clear guidelines for donor screening, ensuring informed consent, and addressing potential risks such as pathogen transmission are essential before these therapies can be widely implemented. Finally, while dietary and lifestyle modifications appear promising, their impact on GM and PCa outcomes has primarily been derived from observational or preclinical studies, highlighting the need for robust clinical trials to confirm their efficacy and long-term benefits.

## 7. Evidence to Date

Much of our current understanding of the GM’s role in PCa progression and treatment response originates from preclinical models and studies in other malignancies. Animal studies have been instrumental in uncovering key mechanisms. For instance, Terrisse et al. demonstrated in murine models that GM depletion through broad-spectrum antibiotics diminished the efficacy of ADT, while FMT from castration-resistant hosts accelerated tumor progression [5,46]. Similarly, Zhong et al. used mouse models to link gut dysbiosis to docetaxel resistance via activation of the IL-6-STAT3 axis, establishing a mechanistic framework for chemotherapy response [48]. Beyond prostate cancer, work by Routy et al. and Gopalakrishnan et al. in epithelial cancers revealed that the GM composition influences ICI responses, suggesting potential for microbial priming to enhance immunotherapy efficacy [56,57].

In contrast, human data remains comparatively limited and heterogeneous. Observational studies, such as those by Fujita et al. and Shin et al., have associated reduced microbial diversity, high-fat Western-style diets, and increased abundance of specific bacterial taxa with prostate cancer risk and severity [12,77]. Clinical investigations into microbiome-modulating interventions, including probiotics and prebiotics, have shown promise in mitigating radiation-induced gastrointestinal toxicity and inflammatory cytokine production in patients undergoing pelvic radiotherapy [71]. However, no large-scale randomized trials have yet evaluated microbiome-targeted therapies in prostate cancer, and much of the clinical evidence is extrapolated from studies in gastrointestinal or other epithelial cancers [73].

These findings underscore a translational gap: while animal models and cross-cancer studies provide valuable mechanistic insights, robust prostate cancer-specific clinical data are still lacking. Bridging this gap will require well-designed prospective trials to validate preclinical findings and establish safe, effective strategies for microbiome modulation in prostate cancer.

## 8. Future Directions and Research Gaps

Research on the GM in prostate cancer remains fragmented by inconsistent methodologies. Differences in sample collection, sequencing technologies, and bioinformatics hinder meaningful cross-study comparisons and translation of findings into clinical practice. Developing standardized protocols for microbiome analysis is critical to establish reproducible, comparable datasets that can support the creation of evidence-based clinical guidelines.

The heterogeneity of prostate cancer and its treatments necessitates individualized approaches to microbiome modulation. Future research should integrate metagenomic, metabolomic, and host immune profiling to design targeted interventions such as tailored probiotics, prebiotics, and engineered microbiomes. Personalized strategies may optimize therapeutic efficacy while minimizing adverse effects, positioning microbiome-based strategies as a viable adjunct to conventional therapy.

Specific taxa such as *Akkermansia muciniphila* have shown promise in enhancing ADT response in preclinical models. Translational studies must now validate these findings in clinical populations, clarifying underlying mechanisms, and exploring the potential for microbiome modulation to improve outcomes across other therapeutic modalities, including immunotherapy and radiotherapy. This targeted approach will help identify the most clinically relevant microbial interventions.

The clinical application of microbiome-targeted therapies requires careful optimization of timing, dosage, and delivery methods. Protocols for interventions such as FMT and probiotic supplementation must balance safety with efficacy, and must be supported by robust donor-screening frameworks and clear ethical oversight. Embedding these strategies into oncology workflows will demand interdisciplinary collaboration between oncologists, microbiologists, and regulatory bodies.

Current knowledge of microbiome-oncology interactions largely derives from preclinical models or studies in other cancers, which may not translate directly to prostate cancer’s unique immunobiology. Dedicated, well-designed clinical trials are needed to validate these findings in prostate cancer populations. Future work should include randomized adjunct trials alongside ADT or radiotherapy, neoadjuvant window-of-opportunity studies before prostatectomy, and prospective longitudinal cohorts incorporating standardized stool and plasma sampling, shotgun metagenomics, metabolomics, and immune profiling at predefined time points. Pragmatic lifestyle trials in men receiving ADT can test implementable diet and exercise programs with embedded microbiome endpoints. Where considered, FMT should begin as a tightly governed safety and feasibility pilot with standardized donor screening and traceability.

## 9. Conclusions

Prostate cancer remains the most common malignancy in men, yet despite decades of research, advanced disease continues to present major therapeutic challenges. In parallel, the GM has emerged as a powerful regulator of systemic immunity, metabolism, and treatment response across multiple cancer types. The convergence of these two fields has revealed compelling mechanistic links between GM composition and prostate cancer progression, as well as its response to androgen deprivation therapy, chemotherapy, radiotherapy, and immunotherapy.

However, much of this evidence stems from preclinical studies and research in other malignancies, leaving critical gaps in prostate cancer-specific knowledge. The data we have are promising but remain insufficient to inform routine clinical practice, underscoring the need for rigorous, large-scale, prostate-focused studies.

Collectively, these findings highlight an urgent need for action. The rapid advancement of microbiome science presents an unprecedented opportunity to integrate microbiome-modulating strategies into prostate cancer care pathways. Achieving this will require standardized analytical methods, the development of robust clinical evidence, and the design of personalized interventions aimed at enhancing treatment efficacy, reducing toxicity, and ultimately improving patient outcomes.

## Figures and Tables

**Table 1:** Microbial species implicated in prostate cancer progression and therapy response. This table summarizes key gut microbial taxa discussed in the review, highlighting their processed roles, underlying mechanisms, and associated treatment contexts based on current literature. ↑ = increased; ↓ = decreased.

Microbial Species	Role	Associated Mechanism	Therapy Context
*Akkermansia muciniphila*	Enhances antitumor activity	Activates CD8+ T cells [27], ↑ IFN-γ and GZMB [27], ↑M1/M2 macrophage Ratio [28]	ADT, Immunotherapy
*Ruminococcus gnavus*	Promotes tryptophan- mediated tumor modulation	Produces AHR ligands from tryptophan [29]	General (Tryptophan metabolism)
*Bifidobacterium bifidum*	Enhances immunotherapy response	↑ IFN-γ production, synergies with immune checkpoint inhibitors [30]	Immunotherapy
*Cutibacterium* spp.	Promotes immunosuppressive environment	↑ IL-17 producing Th17 Cells [31], ↑ Treg Infiltration [31]	General Inflammation
*Faecalibacterium* spp.	Pro-inflammatory in cancer context	Activates NF-κB [32]; ↑ pro-inflammatory cytokines [32]	Inflammation, Colorectal cancer (linked)
*Prevotella* spp.	Associated with inflammation and dysbiosis	Enriched in prostate cancer; ↑ intestinal permeability, inflammation [15]	Inflammation, PCa association
*Lactobacillus* spp.	Reduces chemotherapy- induced toxicity	↑ Mucin secretion [33], ↓ IL-6 and TNF-α, anti-inflammatory effects [33]	Chemotherapy, Radiotherapy
*Alistipes onderdonkii*	Radioprotective	↓ oxidative stress [34]; ↑ mucosal barrier integrity [34]	Radiotherapy
*Mycoplasma hyorhinis*	Reduces efficacy of gemcitabine	Metabolizes gemcitabine into inactive form [22]	Chemotherapy

## Data Availability

No new data were created or analyzed in this study. Data sharing is not applicable to this article.

## Abbreviations

A. muciniphila	Akkermansia muciniphila
ADT	Androgen deprivation therapy
AR	Androgen receptor
AHR	Aryl hydrocarbon receptor
CRPC	Castration-resistant prostate cancer
Dehydroepiandrosterone	DHEA
FMT	Fecal microbiota transplantation
GI	Gastrointestinal
GZMB	Granzyme B
GM	Gut microbiome
HFD	High-fat diet
HSPC	Hormone-sensitive prostate cancer
ICI	Immune checkpoint inhibitors
IGF-1	Insulin-like growth factor 1
IL-2	Interleukin-2
IL-6	Interleukin-6
IL-12	Interleukin-12
LPS	Lipopolysaccharide
mTOR	Mechanistic target of rapamycin
NK	Natural killer
PAMPs	Pathogen-associated molecular patterns
PRRs	Pattern-recognition receptors
PD	Pharmacodynamics
PK	Pharmacokinetics
PTEN	Phosphatase and tensin homolog
PI3K	Phosphatidylinositol 3-kinase
PAHs	Polycyclic aromatic hydrocarbons
PIA	Proliferative inflammatory atrophy
PIN	Prostatic intraepithelial neoplasia
PSA	Prostate specific antigen
SCC	Prostatic small cell carcinoma
AKT	Protein kinase B
RT	Radiotherapy
SCFAs	Short-chain fatty acids
TPH1	Tryptophan hydroxylase 1
TNF	Tumor necrosis factor
TME	Tumor-promoting microenvironment
